# Photoluminescence Characteristics of Zinc Blende InAs Nanowires

**DOI:** 10.1038/s41598-019-54047-8

**Published:** 2019-11-27

**Authors:** E. A. Anyebe, M. Kesaria

**Affiliations:** 1 Federal University of Agriculture, Makurdi, PMB 2373 Nigeria; 2 School of Physics and Astronomy, Cardiff University, Cardiff, UK

**Keywords:** Nanowires, Characterization and analytical techniques

## Abstract

A detailed understanding of the optical properties of self-catalysed (SC), zinc blende (ZB) dominant, nanowires (NWs) is crucial for the development of functional and impurity-free nanodevices. Despite the fact that SC InAs NWs mostly crystallize in the WZ/ZB phase, there are very limited reports on the photoluminescence (PL) properties of ZB InAs NWs. Here, we report on the PL properties of Molecular Beam Epitaxy grown, SC InAs NWs. The as-grown NWs exhibit a dominant band to band (BtB) peak associated with ZB, InAs with an emission energy of ~0.41 eV in good agreement with the band gap energy of ZB InAs and significantly lower than that of the wurtzite phase (~0.48 eV). The strong BtB peak persists to near room temperature with a distinct temperature-dependent red-shift and very narrow spectral linewidth of ~20 meV (10 K) which is much smaller than previously reported values. A narrowing in PL linewidth with increasing NWs diameter is correlated with a decline in the influence of surface defects resulting from an enlargement in NWs diameter. This study demonstrates the high optical property of SC InAs NWs which is compatible with the Si-complementary metal-oxide-semiconductor technology and paves the way for the monolithic integration of InAs NWs with Si in novel nanodevices.

## Introduction

III-V Semiconductor nanowires (NWs) are promising building blocks for novel electronic and optoelectronic devices including, mid-infrared lasers and photodetectors^1–7^. Among them, InAs NWs have attracted enormous interest owing to their narrow direct bandgap, small electron effective mass and high electron mobility. The high ballistic injection velocity of InAs makes it suitable for applications in nanowire field effect transistors (FET)^8–10^ and low power devices such as tunnel FETs since high currents can be achieved in InAs heterojunction^11,12^. InAs is also suitable for methane sensing due to its peak photoresponse of ~3.4 µm at room temperature. In order to avoid the incorporation of unwanted metal impurities, the self-catalyzed growth process is favourable, mostly for the monolithic integration of III-V NWs with the well-established Si platform in functional, impurity-free optoelectronic devices. The Photoluminescence (PL) properties of wurtzite (WZ) phase [WZ dominant^13–15^ and highly polytypic (50% WZ)^16^] InAs NWs has been extensively investigated, mostly realized via the Au-assisted growth technique. However, there are very limited reports of investigations of the optical properties of their ZB counterparts. Sun *et al*.^17^ reported the first PL properties with valuable insight into the optical properties of Au-catalyzed WZ and ZB InAs NWs. The PL properties of ZB InAs NWs grown via the catalyst-free route has been investigated in comparison to an InAs epilayer^18^. However, given the huge importance of binary InAs NWs, it is essential to undertake further PL studies to provide better understanding of its optical properties. More so, the ZB structure is the most predominant phase of InAs NWs grown via the self catalyzed route^19–21^ and to the best of our knowledge there has been no report of the optical properties of Self catalyzed (SC) ZB, InAs NWs. An understanding of the optical properties and the mechanisms of recombination of such SC InAs NWs is crucial for developing functional and impurity-free optoelectronic devices.

In this letter, the detailed PL studies of ZB, In-catalyzed InAs NWs directly grown on bare Si (111) substrates by molecular beam epitaxy (MBE) via an In-droplet assisted technique is reported. The use of pre-deposited In-droplets as catalyst allows for investigation of the optical properties of the NWs with minimal extrinsic contributions.

## Method and Results

InAs NWs were grown on bare Si(111) substrates by MBE via an Indium droplet-assisted growth technique as described previously^19^ under As-rich conditions (Beam equivalent pressure of ~10^−6^ and 10^−7^ mbar for As and In respectively) at a growth temperature of 440–500 °C for about 25 and 144 min for samples α and γ, respectively. Prior to NWs growth, the Si substrates were first dipped in 12% hydrofluoric acid solution for 3 min to remove the native oxide, then immediately loaded into the MBE system and outgassed at 650 °C for >3 hours. The growth was terminated by closing both In and As shutters, simultaneously. The surface morphology of the NWs were investigated by a LEO 1530 Gemini FEG scanning electron microscope (SEM) working at 15 kV. The bulk InAs is a 6 µm thick InAs thin film grown on InAs substrate at 480 °C at a growth rate of 1 ML/s.

To perform PL measurements, the InAs NWs samples were first mounted on a copper cold finger and then inserted in an oxford instrument continuous flow cryostat filled with helium gas to allow for thermal contact. Liquid helium was used for cooling down the samples from 300 to 4 K using a Bentham temperature controller. A spectra-physics model 2011 Ar + ion laser (514 nm) was used as the excitation source while a liquid N_2_ cooled InSb photodiode detector was used for the detection of PL signal from the samples. The excitation spot size of the Ar + ion laser was about 1 mm^2^. A lock-in amplifier and an optical chopper were used to suppress unwanted noise.

**Figure 1:**
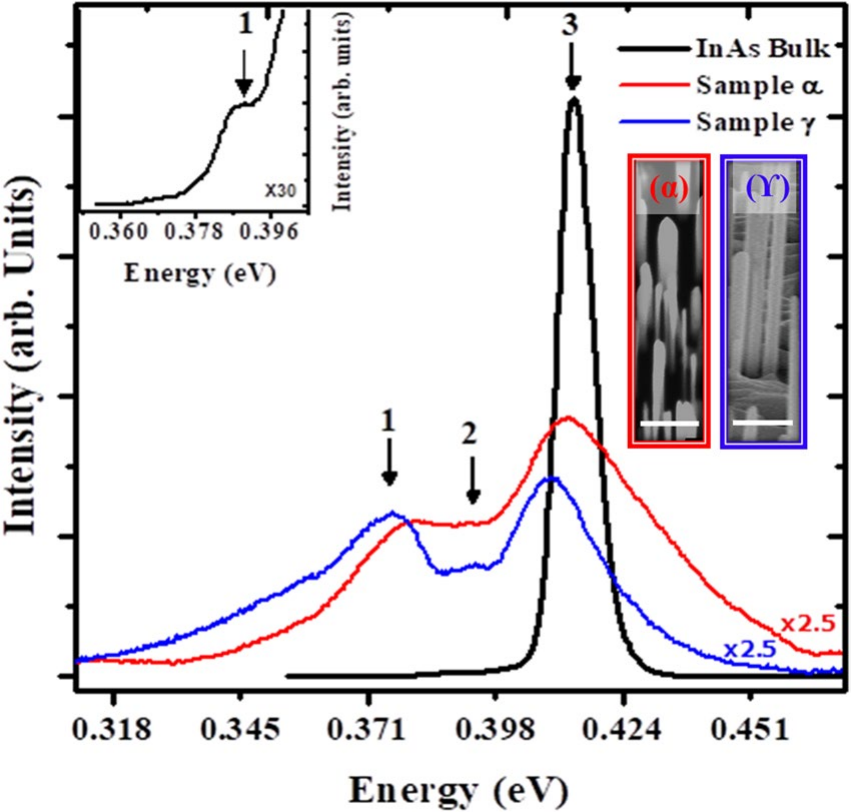
PL spectra of InAs NWs samples at 10 K showing multipeak emissions with peaks 1, 2 and 3 corresponding to deep impurity/defect related, donor-acceptor-pair and band-to-band emissions, respectively. The PL spectrum of InAs bulk is also shown for comparison with Peak 1 magnified and shown in the inset for clarity. Tilted SEM images of as-grown InAs NWs samples α and γ is also shown in the inset (The scale bar for both images is 500 nm).

Figure 1(inset) shows the scanning electron microscopy (SEM) images of as-grown InAs NWs samples α and γ with lengths of 0.90 ± 0.28 µm and 3.82 ± 0.99 µm and diameters of 62.51 ± 26.00 nm and 76.57 ± 6.36 nm respectively. It should be noted that, for each sample, over 70% of measurable NWs were used for the determination of the geometry (length & diameter) and error bars using Gaussian approximations. The error bars in the length & diameter of the NWs is expressed as the deviation from the mean geometry of normally distributed NWs (Further details on how NWs geometry and error bars were obtained are available in the supplementary material). Typical low temperature (10 K) PL spectra of as-grown InAs NWs and a bulk InAs reference is shown in Fig. 1. As expected, the intensity of the InAs bulk is higher than that of the InAs NWs due to the thickness (6 µm) of the MBE grown InAs epilayer on InAs substrate. As can be seen, the InAs NWs exhibit a multi-peak emission which can be resolved into a series of three emission peaks labelled as 1, 2 and 3 for both samples α & γ and the InAs bulk. The PL energies of the InAs NWs are assigned to various transitions as summarized in Table 1 along with that of the InAs epilayer. In order to derive detailed information including the number, intensity, position and width of the PL peaks from the PL spectra, they were fitted with Gaussian approximations (Further information of how the peaks were obtained using Gaussian approximation is provided in the supplementary material).

**Table 1:** Assigned low temperature (10 K) PL emission energies of self-catalyzed InAs nanowires grown on Si in comparison with the values of bulk InAs (all energies are in eV).

Sample	(Peak 1) Deep Impurity/Defect Related	(Peak 2) Donor- Acceptor pair	(Peak 3) Band-to-band
α	0.381^22,24^	0.389^26,27^	0.414^8,23,25^
γ	0.372^17,23^	0.397^17,37^	0.409^9^
Film	0.388^22,24^	—	0.414^8,23,25^

Peaks 1 and 2 are assigned to the deep impurity or defect related (IDR)^17,22–24^ and donor acceptor pair (DAP)^17,25–28^ emissions, respectively while the dominant peak 3 is associated with band-to-band (BtB) transitions. It is worthy to note that the InAs epilayer also displays the IDR peak observed in as grown NWs (inset of Fig. 1). Significantly, the two samples clearly show a dominant BtB emission (peak 3) at ~0.410 eV associated with ZB InAs^22,24,29^ which is consistent with the InAs epilayer (Fig. 1) and in good agreement with the bulk ZB-InAs band gap energy^30^ but significantly lower than the WZ-InAs bandgap (~0.480 eV)^15^ as well as the WZ-dominated (~0.455 eV)^31^ and mixed WZ/ZB-phase InAs NWs^16^. A Previous PL study by Sun *et al*.^17^ carried out on randomly oriented ZB dominant InAs NWs revealed multiple PL signatures assigned to above the InAs band gap, neutral-donor-bound exciton & free-exciton recombination and finally deep impurity or defect-related acceptor. No strong and distinct band edge emission was identified in the noisy PL spectrum due to the poor crystal quality of the sample. A PL peak emission of 0.445 eV was recently reported for ∼50 nm sized InAs NWs by Koblmuller *et al*.^18^. The blue shift in band edge emission in their work in comparison to the current work is understandable owing to the smaller NWs diameter resulting in stronger quantum confinement effect (QC). In their work, a shoulder at around ∼0.50 eV was observed on the high energy side of the PL peak which they believed could have arisen from band transitions associated with occasional WZ segments. Such WZ segments could possibly contribute to the blue shift of their PL spectrum considering the large WZ-InAs bandgap. Thus, the observed PL peak energies of as-grown samples that are below the band gap value of the phase-pure WZ InAs reveal the ZB dominant phase of our NWs. However, we cannot totally exclude the possibility of contribution from the type-II band alignment transition resulting from the presence of small WZ insertions in as-grown NWs (more detailed discussion on this to follow in a subsequent section). It is worthwhile mentioning that the nano-scale geometry of the NWs may result in QC effect. Whereas the PL peak of sample γ (~77 nm) is positioned at a lower energy of 0.409 eV, the peak associated with the BtB transition of sample α (~65 nm) was blue shifted to 0.414 eV. The observed small blue shift of sample α could be attributed to QC effect owing to the relatively small diameters of the NWs (only slightly larger than the Bohr radius of InAs at ∼34 nm). This diameter-dependent band gap is in good agreement with previous report^22^ and confirms the onset of QC for InAs NWs at a fairly large.

NW diameter (>60 nm) due to the very small electron effective mass of InAs. QC effect is expected to be more significant for smaller NWs diameter^18^. As can be observed from Table 1, the NWs emission of the quasi pure ZB InAs sample γ (BtB peak = 0.409 eV) is slightly red shifted with respect to that of the bulk ZB InAs (BtB peak = 0.414 eV). The observed redshift might be due to the different carrier densities in NWs and bulk. InAs being a degenerate narrow band-gap semiconductor usually possesses a higher n-type carrier density in comparison to their bulk counterpart. Koblmuller *et al*.^7^ observed a similar redshift in NWs structures as opposed to the bulk samples. Similarly, Sonner *et al*.^31^ reported a red shift in low-temperature bandgap energy with increasing n-type carrier concentration.

Intriguingly, whereas the peak α_3_ display a large spectral line width (full width at half maximum) of ~35 meV, peak γ_3_ exhibits a record narrow spectral linewidth of ~ 20 meV which is closer to that of the InAs reference peak 3 (~7 meV) and smaller than the commonly reported values (29–34) meV^17,22^ which suggests an improved quality of sample γ. We attribute the narrowing in PL linewidth with increasing NWs diameter to a decline in the influence of surface defects owing to an enlargement in NWs size and a reduction in surface to volume ratio of as-grown NWs^15,22^. A size-related decrease of WZ insertions in ZB has been excluded as it will be shown later. To exclude the contributions to the PL emissions from the clusters grown alongside the NWs, PL measurements were exclusively performed on a sample with only InAs islands and no detectable PL emission was observed. This is understandable given the poor material quality of the islands resulting from the high density of dislocations and anti-phase domains. This suggests that it is less likely that emissions from the clusters grown alongside the NWs contributes to the observed PL emission.

**Figure 2:**
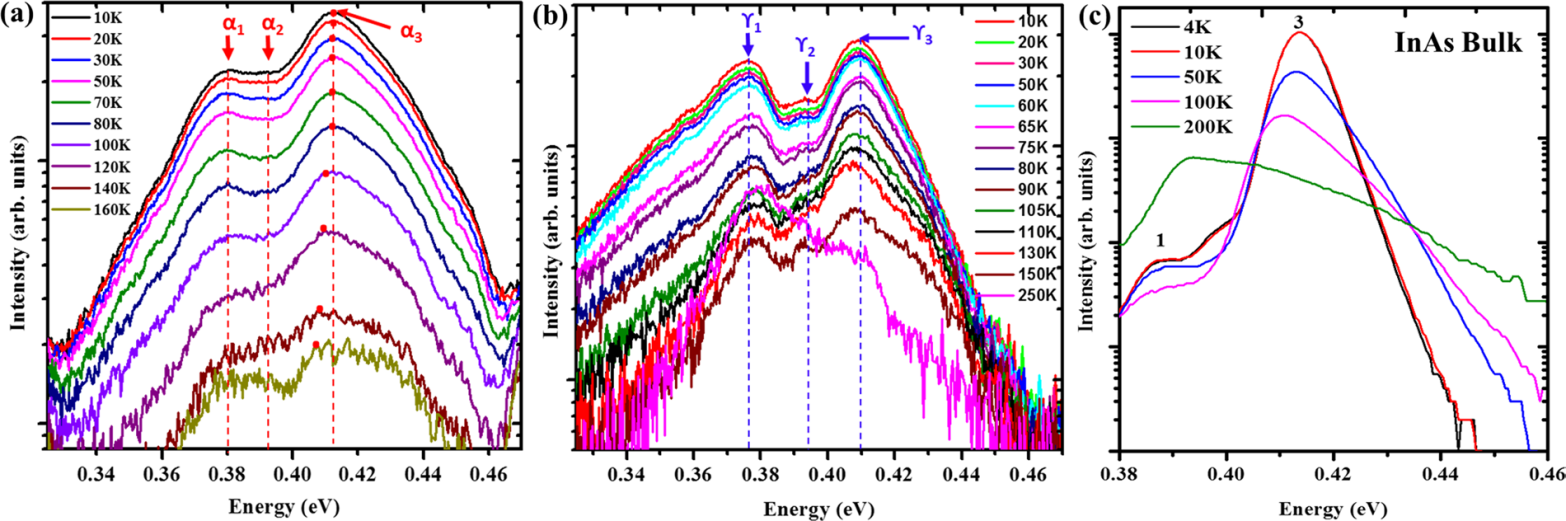
Temperature-dependent PL emission spectra of InAs nanowires samples α (**a**) and γ (**b**) compared with an InAs epilayer (**c**). The peaks identified as (α_1_, & γ_1_), (α_2_, & γ_2_) and (α_3_ & γ_3_) correspond to the deep impurity/defect related, donor-acceptor-pair and band-to-band emissions of samples α and γ, respectively. Peaks 1 and 3 are associated with the deep impurity/defect related and band-to-band emissions of the InAs bulk sample.

To elucidate the origins of the various transitions in as-grown NWs, temperature and power-dependent PL measurements were performed. The temperature-dependent PL spectrum of sample α is shown in Fig. 2(a). Peaks α_**1**_ and α_**2**_ show no obvious shift with increasing temperature which agrees with our assignment of the peaks to IDR and DAP transitions respectively. Peak α_3_ shows an obvious red-shift of about 5 meV with a rise in temperature from 10 to 160 K, while the PL intensity decreases with increasing FWHM from about 35 to 57 meV (10–120 K). A similar behaviour was also observed for sample γ. As shown in Fig. 2(b), peak γ_3_ shows a red-shift of 6 meV with increasing temperature (10–250 K) while the FWHM increases from ~20 to 42 meV. This is in good agreement with the generally observed monotonous band gap shrinkage with increasing temperature in semiconductors, which provides convincing evidence that peak γ_3_ is associated with the BtB emission. Interestingly, that there was no observable S-shape” behaviour (blueshift/redshift) of the PL peak energy with increasing temperature which is a signature of localized states induced by potential variations from WZ/ZB mixed stacking as previously reported for polytypic InAs NWs^31,32^. This indicates that there were no significant contributions from type II band alignment to the PL emissions.

The energy of the peaks γ_**1**_ and γ_**2**_ [Fig. 2(b)] exhibits a temperature independence over the investigated temperature range (10–250 K) which is typical for defects or impurity related emissions^24^. Similarly, the temperature-dependent PL spectrum of bulk InAs epilyer (Fig. 2c) shows a clear red-shift in BtB (peak 3) PL energy with no-shift in peak 1 position. Interestingly, although the NWs are unpassivated, peak γ_3_ persists up to a high temperature of 250 K, which is higher than that of sample α (160 K) as well as previously reported InAs NWs (110–200 K)^13,17,22^. Since peak 3 (BtB) is the dominant emission, we plotted the PL energy of the NWs as a function of temperature.

**Figure 3:**
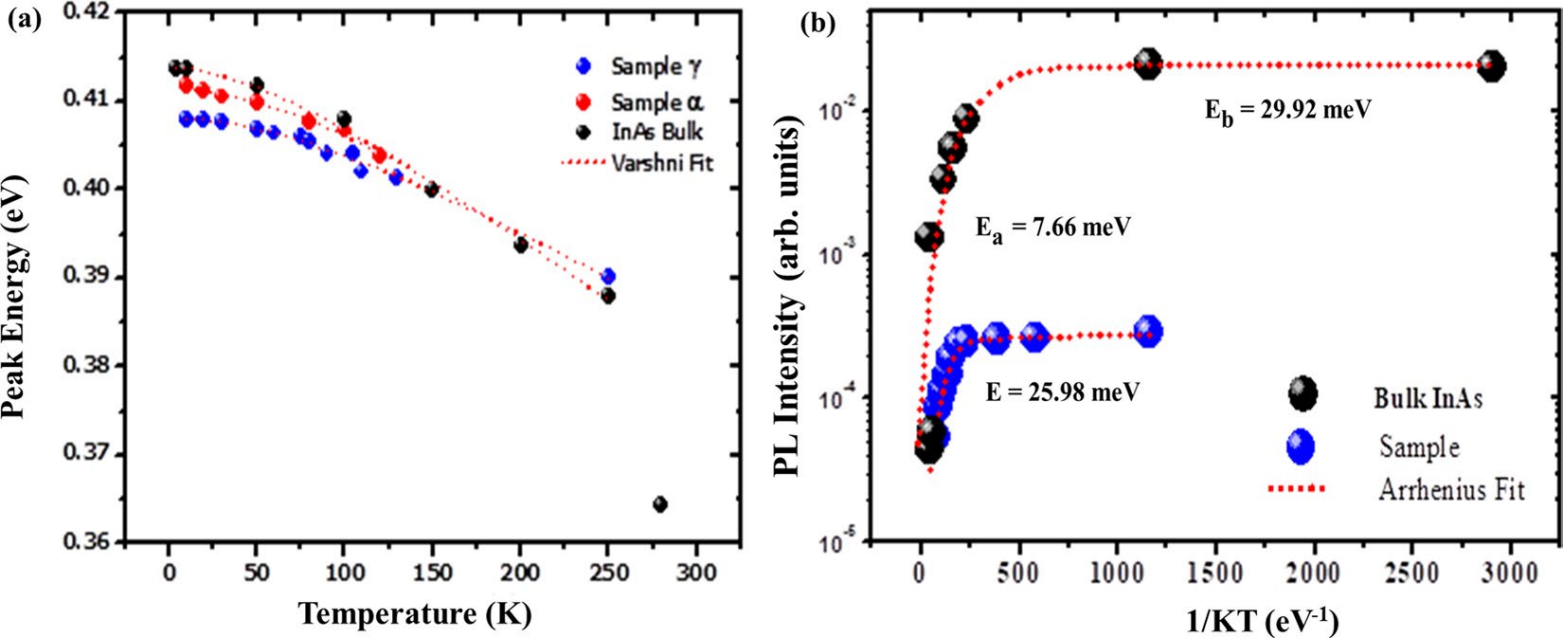
(**a**) Variation of PL peak energy of InAs nanowires (samples α and γ) and InAs bulk as a function of Temperature. The dotted curves represent varshni fits. (**b**) The Arrhenius plot of integrated PL intensity versus 1/KT for InAs epilayer and sample γ. The dotted curves representthe best arrhenius fit while E_a_ and E_b_ indicate the obtained activation energies at high and low temperatures.

As shown in Fig. 3a, the temperature dependent red-shift in PL peak energy of as-grown NWs is much weaker in comparison to the bulk InAs. This is consistent with previous reports^22,31^ and associated with the presence of an electron accumulation layer in the NWs surface resulting in band bending. Worthy of note is the slow and gradual decrease in peak energy of the two samples which is significantly different from the rapid energy decrease (30–35 meV) typical of type-II transition related transitions^16^ (for a similar temperature interval) due to the minimization of band bending as a result of thermally-induced transfer of electrons from the ZB to WZ conduction band of InAs at higher temperatures. It is well-known that the dependence of semiconductor bandgap energy on temperature follows the Varshni empirical formula^33^:

$${{\rm{E}}}_{{\rm{g}}}({\rm{T}})={{\rm{E}}}_{0}-{{\rm{AT}}}^{2}/(B+T)$$

where E_0_ is the energy gap at 0 K, T is the temperature, A and B are associated with the thermal expansion coefficient and the Debye temperature respectively. The extracted values for coefficients “A” were (1.98 ± 2.23, 1.28 ± 0.18 and 1.57 ± 0.27) × 10^−4^ meV/K and (263 ± 431, 189 ± 59 & 119 ± 61) K, for B corresponding to samples α, γ and bulk InAs respectively.

To evaluate the temperature PL quenching process of SC InAs NWs, the integrated PL intensities of peak 3 (BtB) were plotted as a function of 1/KT in Fig. 3(b) with Arrhenius simulation (dotted lines) using Arrhenius equation:

$${\rm{I}}({\rm{T}})=(I(0)/\,[1+C{e}^{-\frac{{E}_{a}}{kT}}+D{e}^{-\frac{{E}_{b}}{kT}}],$$

where I (0) is the spectral intensity at low temperatures, C and D measures the quenching mechanism, K is the Boltzmann constant and T is the temperature, while E_a_ and E_b_ denote the thermal activation energies at high and low temperatures respectively. Activation energies of 7.66 ± 26.22 meV & 29.92 ± 55.97 meV for the InAs epilayer within the regime of high and low temperatures respectively ascribed to exciton dominated emission and electron-hole plasma emission respectively. On the other hand, an activation energy of 25.98 ± 20.99 meV was extracted for the as-grown sample γ, corresponding to electron-hole plasma emission^17^.

**Figure 4:**
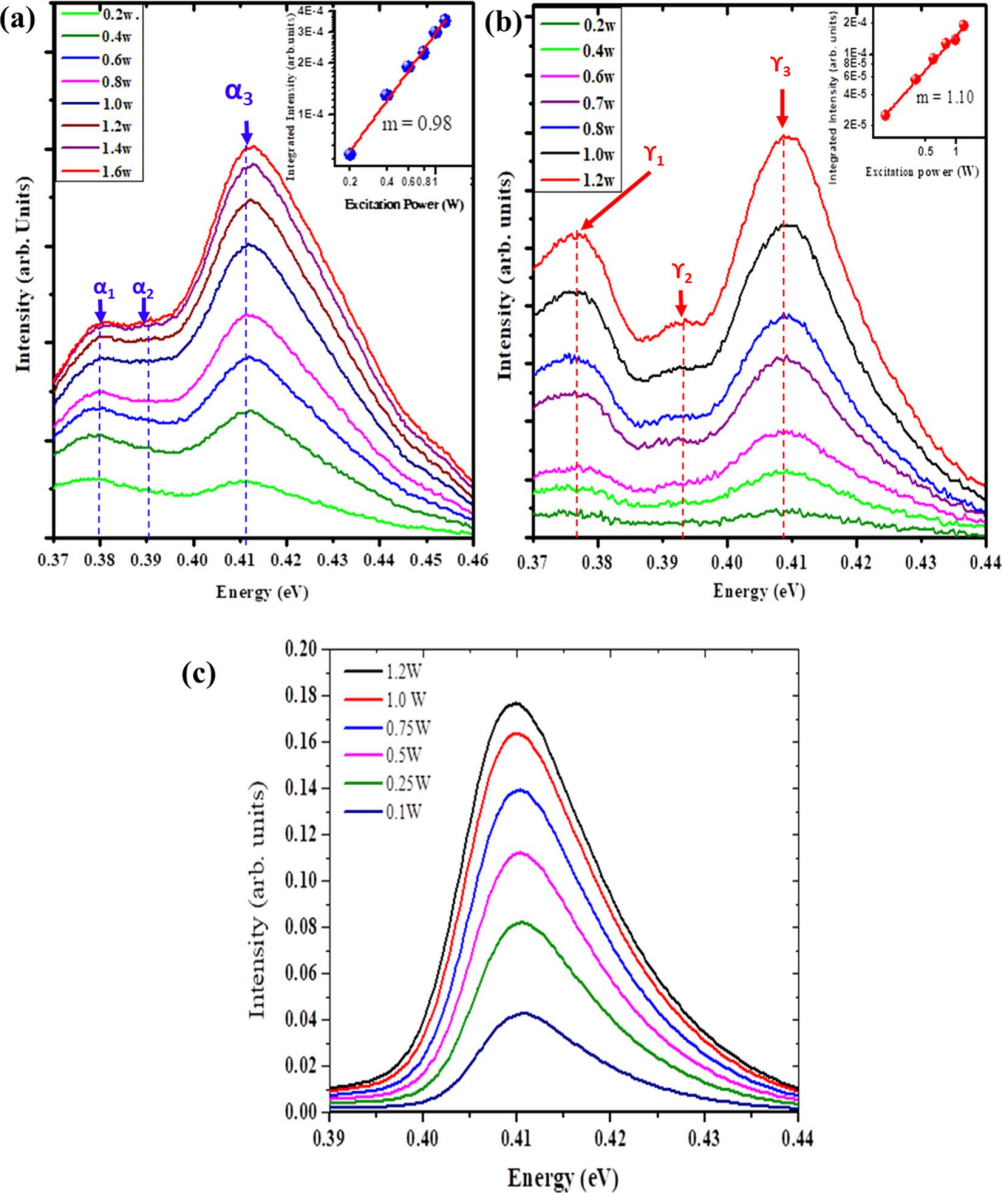
Power-dependent PL emission spectra of InAs nanowires samples α (**a**) and γ, (**b**) measured at 4 K compared to an InAs bulk epilayer (**c**). The inset shows the dependence of PL intensity on excitation power. The peaks identified as (α_1_, & γ_1_), (α_2_, & γ_2_) and (α_3_ & γ_3_) correspond to the deep impurity/defect related, donor-acceptor-pair and band-to-band emissions of samples α and γ, respectively.

Figure 4 shows the 4 K PL spectra measured under various laser powers for the InAs NWs samples (α and γ) and InAs epilayer. The insets show that the integrated intensity (I) of the BtB emissions are linearly related to the excitation power (P). Fitted power factors of m = 0.98 and 1.10 (~1) were obtained for the investigated samples α and γ respectively in accordance with the relation I (P) ~ P^m^. A small blue shift of ~4 meV was observed for the BtB peak of sample α which can be attributed to a band filling of photocarrier^15,34^. However, there was no significant change (~0.2 meV) in the BtB peak emission of sample γ (γ_**3**_) which is consistent with a previous study^17^. The near absence of excitation power dependence of the PL peak positions can be attributed to a weak band filling effect obscured by a slight broadening of the PL peak^22,34^ which could be largely attributed to band bending effect. Such an insensitive behaviour to variations in excitation power was also observed in the InAs epilayer sample (Fig. 4c) and has being previously reported for high quality NWs^35^.

It is worthy to note that the presence of WZ insertions in ZB dominant crystals could possibly lead to contributions from Type II related emissions to the PL peaks. This is particularly significant due to the higher bandgap energy of WZ InAs, with a difference of 40–66 meV^36–38^ in relation to their ZB counterpart. It has been shown^14^ that a type-II quantum well (QW) related emission from WZ and ZB sections of polytypic crystals could result in a large blue-shift with significant broadening as a function of excitation power. A large blue shift of 15–30 meV with distinct peak broadening was observed with increasing excitation power (0.01–0.5 W) and attributed to band bending^14^. Electrons and holes confined within the type-II QW of the ZB structure and optically excited, the electrons transit from the confined quantum ground state in the ZB region to the top of the WZ valence band. With an increase in excitation power, band bending and state filling of electrons in the ZB section and holes in the WZ regions results in distinct blue-shift with significant broadening^14^. A similar behaviour was also observed in polytypic InAs^16^, GaAs^39^ and InP NWs^40,41^. However, we did not observe this large blue-shift for the investigated excitation power (0.2–1.6 W). This can be correlated to the predominant ZB phase of the two samples leading to a dominant ZB, BtB transition. This explains the exclusion of a size-related decrease of WZ insertions in ZB in relation to the diameter dependent line width. Significantly, unlike the highly polytypic InAs^16^ (50% WZ phase) NWs which showed a high temperature peak at ~0.425 eV associated with the BtB transition of the WZ phase, there was no observable peak within this region which further demonstrates that the contributions of the WZ segments (WZ Phase fractions) to the PL emissions is negligible. Consequently, the observed α_**3**,_ γ_**3**_ peaks are ascribed to the BtB transitions of the ZB phase of InAs NWs resulting from the predominant ZB crystals of as-grown NWs. The absence of type II related blue shift in the IDR (α_**1**,_ γ_**1**_) and DAP (α_**2**,_ γ_**2**_) peaks of samples α and γ (Fig. 4a,b) further corroborates this assertion. This confirms the narrow WZ insertions of as-grown samples as revealed by transmission electron microscopy analysis^19^ and demonstrates that the contributions of Type II related transitions is strongly dependent on the size of the WZ Phase fractions which is highly dependent on growth conditions^42–45^ and NWs geometry^45,46^. Therefore, it can be inferred that the BtB transitions would be the predominant emission for a ZB dominant crystal with low WZ Phase fractions (with the possibility of insignificant contributions from Type II related emissions). Conversely, the contributions of type-II transitions are more likely to be significant for a crystal with large WZ Phase fractions^16^.

## Conclusion

In Summary, the optical properties of self-catalyzed, ZB InAs NWs grown via an indium droplet-assisted technique is reported. The NWs exhibit a multi-peak emission including a dominant band to band (BtB) peak associated with the ZB dominant, self-catalyzed InAs NWs. The contributions from the Type II related emissions is weak due to the low WZ-phase fractions in as-grown NWs. The strong BtB peak persists to near room temperature with a distinct temperature-dependent red-shift and very narrow spectral linewidth of ~20 meV (10 K). This study demonstrates the high optical quality of ZB dominant, self-catalyzed InAs NWs with promising applications in optoelectronic devices.

## Supplementary information

Supplementary information
