# Supplementary material for: Photoluminescence Characteristics of Zinc Blende InAs Nanowires
Source: Sci Rep. 2019 Nov 27;9:17665. doi: 10.1038/s41598-019-54047-8 (PMC6881312; doi:10.1038/s41598-019-54047-8)
Supplement: Supplementary file 1 — Supplementary information [file 41598_2019_54047_MOESM1_ESM.pdf]

Photoluminescence Characteristics of Zinc Blende InAs Nanowires

E.A. Anyebe[1] and M. Kesaria[2*]*

[1]Federal University of Agriculture, Makurdi, PMB 2373, Nigeria.

[2]School of Physics and Astronomy, Cardiff University, Cardiff, UK

**corresponding authors:* ezeanyabe@hotmail.co.uk; kesaria123@gmail.com

Supplementary Material

The geometry and uncertainty of as grown InAs NWs and bulk were determined using Gaussian approximations. Typical Gaussian Fits of the Length and diameter of InAs NWs (sample α) are shown in Figure S1a and b respectively.

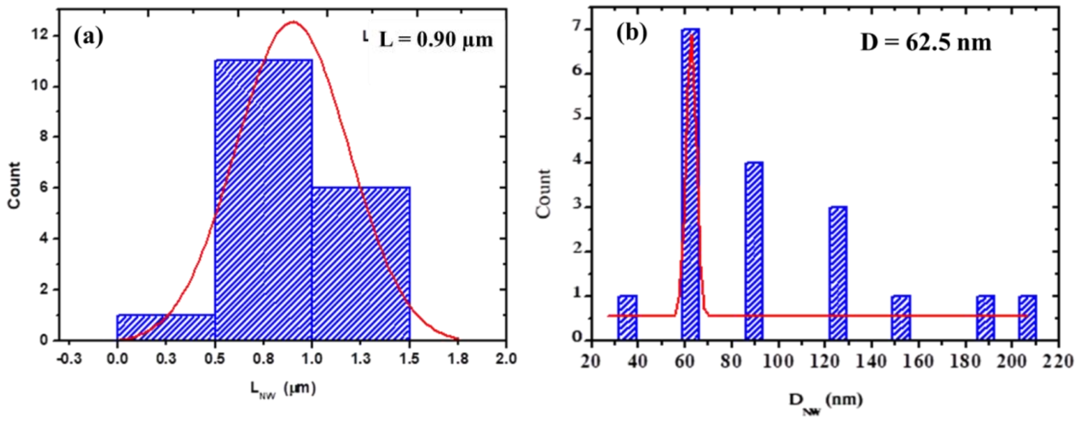

Figure S1: Typical Gaussian Fit of the Length and diameter of InAs NWs (sample α)

Figure S2a typically shows how peaks γ_1, γ_2 and γ_3 of sample γ were obtained using Gaussian Fit. Similarly, the Gaussian Fitting for the InAs Bulk is depicted in Figure S2b. However, it should be noted that it was difficult fitting the low intensity low and high energy tails of samples gamma and InAs bulk consequently, these peaks were not included in the Gaussian Fit.

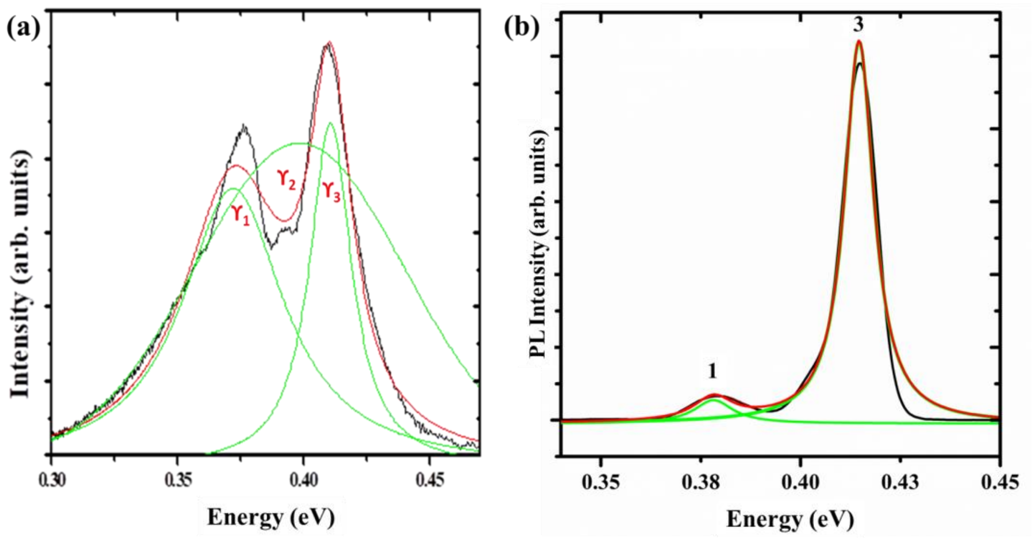

Figure S2: Gaussian Fit demonstrating how peaks γ_1, γ_2 and γ_3 of sample γ were obtained (a). The Gaussian Fit for the InAs Bulk is also shown (b)
